# DEVELOPMENT OF ADENOCARCINOMA AFTER RADIOFREQUENCY ABLATION OF BARRETT’S ESOPHAGUS ASSOCIATED TO FUNDOPLICATION AND SUPPRESSION-DUODENAL DIVERSION PROCEDURE: A LESSON TO BE LEARNED

**DOI:** 10.1590/0102-672020230068e1786

**Published:** 2023-12-18

**Authors:** Italo Braghetto

**Affiliations:** 1 University of Chile, Faculty of Medicine, Department of Surgery, Hospital “Dr. José J. Aguirre”– Santiago de Chile, Chile.

**Keywords:** Barrett Esophagus, Endoscopy, Radiofrequency Ablation, Argon Plasma Coagulation, Fundoplication, Gastrectomy, Esôfago de Barrett, Endoscopia, Ablação por Radiofrequência, Coagulação com Plasma de Argônio, Fundoplicatura, Gastrectomia

## Abstract

Despite endoscopic eradication therapy being an effective and durable treatment for Barrett’s esophagus-related neoplasia, even after achieving initial successful eradication, these patients remain at risk of recurrence and require ongoing routine examinations. Failure of radiofrequency ablation and argon plasma coagulation is reported in 10–20% of cases.

## RESUMO

Apesar de a terapia de erradicação endoscópica ser um tratamento eficaz e durável para a neoplasia relacionada ao esôfago de Barrett (BE), mesmo após a erradicação inicial bem-sucedida, esses pacientes permanecem em risco de recorrência e requerem exames de rotina contínuos. A falha na ablação por radiofrequência e na coagulação com plasma de argônio é relatada em 10–20% dos casos.

Recently, it was published the paper “Suppression-duodenal diversion procedure for long-segment Barrett´s esophagus: early and long-term outcome”^1^. During the postoperative follow-up, eradication of dysplasia was obtained, as shown in Table 5 of this paper. However, in one patient initially presenting low grade dysplasia, after 5 years of follow-up, the presence of adenocarcinoma at distal esophagus was confirmed histologically, probably remaining buried cells (Table 1)^1^. The complete assessment included computed tomography (CT) scan, positron emission tomography (PET) scan, and endo-sonography, affirming a T1b esophageal adenocarcinoma. The definitive treatment indicated for this patient was esophagectomy with colon interposition.

**Table 1:** Histologic results after treatment with argon plasma coagulation or radiofrequency ablation in patients with intestinal metaplasia alone or with dysplasia. The results were observed during follow-up^1^.

			APC	RFA	p-value
			n=21 (%)	n=31 (%)	
Intestinal metaplasia					
	Before treatment		20 (95.2)	25(80.6)	<0.21
	After treatment				
		Total initial eradication	14 (66.6)*	17 (54.8)*	<0.02
		Residual island	6 (28.6)†	8 (25.8)†	<0.02
		Reappearance during follow-up	1 (5)†	4 (12.9)†	>0.37
		Progression to LGD	0	0	
Intestinal metaplasia with LGD					
	Before treatment		1 (4.8)	6 (19.4)	>0.21
	After treatment				
		Total initial eradication	1 (100)*	4 (66.6)*	>0.37
		Residual island of IM	0	2‡	1.0
		Reappearance IM during follow-up	1†	1†	>0.4
		Reappearance LGD during follow-up	0	0	
		Progression to HGD during follow-up	0	0	
		Progression to adenocarcinoma	0	1§	

* Eradication with regression to carditis

† Treated with repeated APC

‡ Submitted to endoscopic submucosal dissection.

§ Submitted to esophageal resection.

APC: argon plasma coagulation; RFA: radiofrequency ablation; LGD: low-grade dysplasia; IM: intestinal metaplasia; HGD: high-grade dysplasia.

This finding encourages a very close follow-up because the risk of reappearance of dysplasia remains and, therefore, it is important to keep attention to this eventual complication that unfortunately, occurred to our patient.

Despite the fact that endoscopic eradication therapy is an effective and durable treatment for Barrett’s esophagus (BE) related neoplasia, even after achieving initial successful eradication, these patients remain at risk of recurrence and require continuous examinations. Failure of radiofrequency ablation (RFA) and argon plasma coagulation (APC) is reported in 10–20% of cases^1,3^.

The incidence rate of BE recurrence, reported by Cotton et al., was 10.8 per 100 person-years (PY) overall (95%CI 7.8–15.0); 8.3 per 100-PY among patients with baseline low-grade dysplasia (95%CI 4.9–14.0); and 13.5 per 100-PY among patients with baseline high-grade dysplasia (95%CI 8.8–20.7)^3^.

The incidence rate of dysplasia recurrence was 5.2 per 100-PY overall (95%CI 3.3–8.2); 3.3 per 100-PY among patients with baseline low-grade dysplasia (95%CI 1.5–7.2), and 7.3 per 100-PY among patients with baseline high-grade dysplasia (95%CI 4.2–12.5)^2,3^.

For other authors, BE recurrence after ablation can occur in almost one-third of patients with baseline dysplastic disease — mostly during the first year after complete eradication of intestinal metaplasia (CEIM)^4,7,9^.

Titi et al. reported the development of subsquamous neoplasia in three patients who were treated with RFA for BE (two developed adenocarcinoma and one developed high-grade dysplasia)^10^. Therefore, patients with BE treated with RFA have a risk of developing subsquamous high-grade dysplasia and adenocarcinoma after successful RFA of BE. In other publications, the development of subsquamous high-grade dysplasia and adenocarcinoma after successful RFA of BE has been also reported. A better prognosis was obtained after combining ablation and proton pump inhibitors^5,11^.

Our idea went even further ahead performing ablation combined with acid suppression-bile diversion surgical procedure. But, a very disappointing experience occurred with this patient previously mentioned. For this reason, follow-up endoscopies every 6 months are a valid alternative, and it is the way to detect early recurrence or progression to dysplastic lesions^6,8^.

This is a great lesson to keep in mind!
